# Propensity Score-Weighted Analysis of Postoperative Infection in Patients With and Without Preoperative Urine Culture

**DOI:** 10.1001/jamanetworkopen.2024.0900

**Published:** 2024-03-04

**Authors:** William J. O’Brien, Marin L. Schweizer, Judith Strymish, Brice F. Beck, Vanessa Au, Jeffrey A. Chan, Madisen Brown, Kamal M. F. Itani, Kimberly C. Dukes, Julia Friberg Walhof, Kalpana Gupta

**Affiliations:** 1 Veterans Affairs (VA) Boston Center for Healthcare Organization and Implementation Research (CHOIR), Boston, Massachusetts; 2 William S. Middleton VA Hospital, Madison, Wisconsin; 3 Department of Medicine, University of Wisconsin-Madison, Madison; 4 VA Boston Department of Medicine, Boston, Massachusetts; 5 Center for Access & Delivery Research and Evaluation (CADRE), Iowa City VA Health Care System, Iowa City, Iowa; 6 Carver College of Medicine, The University of Iowa, Iowa City; 7 VA Boston Health Care System Department of Surgery, Boston University and Harvard Medical School, Boston, Massachusetts

## Abstract

**Importance:**

Although recent guidelines recommend against performance of preoperative urine culture before nongenitourinary surgery, many clinicians still order preoperative urine cultures and prescribe antibiotics for treatment of asymptomatic bacteriuria in an effort to reduce infection risk.

**Objective:**

To assess the association between preoperative urine culture testing and postoperative urinary tract infection (UTI) or surgical site infection (SSI), independent of baseline patient characteristics or type of surgery.

**Design, Setting, and Participants:**

This cohort study analyzed surgical procedures performed from January 1, 2017, to December 31, 2019, at any of 112 US Department of Veterans Affairs (VA) medical centers. The cohort comprised VA enrollees who underwent major elective noncardiac, nonurological operations. Machine learning and inverse probability of treatment weighting (IPTW) were used to balance the characteristics between those who did and did not undergo a urine culture. Data analyses were performed between January 2023 and January 2024.

**Exposures:**

Performance of urine culture within 30 days prior to surgery.

**Main Outcomes and Measures:**

The 2 main outcomes were UTI and SSI occurring within 30 days after surgery. Weighted logistic regression was used to estimate odds ratios (ORs) for postoperative infection based on treatment status.

**Results:**

A total of 250 389 VA enrollees who underwent 288 858 surgical procedures were included, with 88.9% (256 753) of surgical procedures received by males and 48.9% (141 340) received by patients 65 years or older. Baseline characteristics were well balanced among treatment groups after applying IPTW weights. Preoperative urine culture was performed for 10.5% of surgical procedures (30 384 of 288 858). The IPTW analysis found that preoperative urine culture was not associated with SSI (adjusted OR [AOR], 0.99; 95% CI, 0.90-1.10) or postoperative UTI (AOR, 1.18; 95% CI, 0.98-1.40). In analyses limited to orthopedic surgery and neurosurgery as a proxy for prosthetic implants, the adjusted risks for UTI and SSI were also not associated with preoperative urine culture performance.

**Conclusions and Relevance:**

This cohort study found no association between performance of a preoperative urine culture and lower risk of postoperative UTI or SSI. The results support the deimplementation of urine cultures and associated antibiotic treatment prior to surgery, even when using prosthetic implants.

## Key Points

**Question:**

Is the practice of preoperative urine culture associated with reduced risk of postoperative urinary tract infection or surgical site infection?

**Findings:**

In this cohort study of 250 389 US veterans who underwent 288 858 surgical procedures, after balancing patient characteristics, there was no significant difference in postoperative infection risk between operations with and without a preoperative urine culture.

**Meaning:**

Findings of this study suggest that preoperative urine culture is a low-value intervention for most surgical patients and should be deimplemented.

## Teaser

This cohort study estimates the probability of receiving urine culture testing and treatment for asymptomatic bacteriuria before noncardiac, nonurologic surgery among veterans at Veterans Affairs medical centers across the US.

## Introduction

Positive urine cultures without symptoms of infection, known as asymptomatic bacteriuria (ASB), are a major factor in inappropriate antibiotic use.^1,2^ The 2019 Infectious Diseases Society of America clinical practice guidelines recommend against testing for and treating ASB in all patients undergoing nonurological surgical procedures.^3^ Similarly, the American College of Physicians considers preoperative urine testing to be a low-value practice.^4^

Despite these guidelines stating that preoperative urine cultures should not be performed, routine preoperative urine culture and antibiotic treatment persist. Prior studies have shown that up to 25% of nonurological surgical procedures are preceded by a screening urine culture, and the rate is almost 50% among certain surgical specialties.^5,6,7^ This practice is also more frequent prior to implant-related procedures.^6^ Testing plays a role in antibiotic treatment; a US study of surgical patients found that antibiotic use was increased in patients with positive urinalysis results despite negative urine cultures.^7^ A separate study demonstrated that after a urine culture is ordered and found to be positive for infection, antibiotic treatment often follows, even if the patient is entirely asymptomatic.^8^

The factors underlying urine culture practices are not well understood. The association between preoperative urine culture and postoperative infection is often confounded by indication (ie, sicker patients get tested and are at higher risk for adverse postoperative outcomes). The risk factors in performance of a urine culture in the first place confound the associations between urine culture and outcomes, such as urinary tract infection (UTI) and surgical site infection (SSI)^9,10^ This confounding leads to uncertainty in the robustness of the published literature.^3^

The goal of this study was to reduce confounding by indication, adjusting for these underlying patient factors using inverse probability of treatment weighting (IPTW). Specifically, we aimed to assess the association between preoperative urine culture testing and postoperative UTI or SSI, independent of baseline patient characteristics or type of surgery.

## Methods

### Study Design and Data Sources

In this retrospective cohort study, we included Veterans Affairs Surgical Quality Improvement Program (VASQIP)–reviewed noncardiac surgical procedures performed from January 1, 2017, to December 31, 2019, at any of 112 US Department of Veterans Affairs (VA) medical centers that perform surgery.^11^ Genitourinary surgical procedures were excluded given that preoperative urine testing is often appropriate for these patients. Patients with any known history of cystectomy (defined by *Current Procedural Terminology* code) were also excluded. To assess the association between ASB and postoperative outcomes, we excluded all patients who had a fever or diagnosis of a UTI in the 30 days prior to surgery. The VA Central Institutional Review Board approved this study and waived the informed consent requirement because risk was minimal. We followed the Strengthening the Reporting of Observational Studies in Epidemiology (STROBE) reporting guideline.

Data on surgical procedures, patient baseline characteristics, and 30-day postoperative outcomes were obtained from the VASQIP, a representative national sample of major surgical procedures abstracted by trained nurses.^12^ The exposure of interest was preoperative urine culture performed within 30 days of surgery, and it was obtained from the Clinical Data Warehouse (CDW) Laboratory Microbiology database. Urine cultures performed on the day of an emergency department visit were excluded because the goal of this analysis was to evaluate routine preoperative urine culture among asymptomatic patients.

We assessed 2 outcomes in the 30-day postoperative period: (1) UTI, which was identified directly from the patient medical record–level data entered into VASQIP, and (2) SSI, which was defined as any VASQIP-assessed superficial, deep wound, and organ/space infection. Both outcomes were designated by a trained reviewer and defined using National Healthcare Safety Network criteria as previously described.^6^

Other covariates that were measured using VASQIP or CDW data included age, sex, Charlson Comorbidity Index (CCI),^13^ American Society of Anesthesiologists Physical Status Classification, VA medical center, preoperative urinary catheterization or retention, any history of prostatectomy, and 3-year history of UTI. Race and ethnicity data were obtained from CDW and reflect the patient electronic medical records. Categories included Black, White, and other (including American Indian or Alaska Native, Asian or Pacific Islander, Hispanic Black, and Hispanic White; these groups were collapsed into a single category because individual sample sizes were small). Race and ethnicity were included in the analysis because there can be variation in treatment on the basis of these factors. Antibiotic data were obtained from the CDW outpatient pharmacy and Barcode Medication Administration tables. Eligible antibiotics were any systemic antibacterial agent administered between 30 days prior to a positive urine culture and 2 days prior to surgery. We excluded antiviral and antifungal agents.

### Statistical Analysis

We constructed a propensity score using IPTW to balance observed baseline characteristics among treatment groups. Weighted logistic regression was then performed to estimate the odds ratios (ORs) for postoperative UTI and SSI, comparing patients who did vs did not have a preoperative urine culture. A second analysis using the same methods was performed among only patients who underwent orthopedic surgery and neurosurgery to focus on surgical procedures with a high frequency of implant-related procedures.

We estimated a propensity score model. Each surgical patient was assigned a probability of preoperative urine culture based on preoperative characteristics (Table 1). These characteristics included age, sex, CCI, VA medical center, Centers for Disease Control and Prevention wound classification, receipt of antibiotics within 30 days before surgery, preoperative urinary catheterization or retention, any history of prostatectomy, and 3-year history of UTI. Probabilities were estimated using XGBoost,^14^ a class of machine-learning algorithm that has been shown in simulation studies to improve covariate balance for binary or multinomial outcomes compared with logistic regression.^15,16,17^ The IPTW weights, which are used to balance baseline characteristics across groups, were calculated as the reciprocal of the probability of receiving the treatment that was actually administered. We stabilized the weights to preserve relative sample size among groups and avoid the need for SE adjustment in the outcome regression.^18^

**Table 1.** Characteristics of Surgical Procedures and Patients Before and After Inverse Probability of Treatment Weighted (IPTW) Balancing

Characteristic	Observed cohort before IPTW				Balanced cohort after IPTW		
	Overall, No. (%)	30-d Preoperative urine culture, No. (%)		SMD	30-d Preoperative urine culture, No. (%)		SMD
		No	Yes		No	Yes	
No. of procedures	288 858	258 474	30 384		258 426.3	30 092.5	
Sex							
Male	256 753 (88.9)	229 948 (89.0)	26 805 (88.2)	0.02	229 716.4 (88.9)	26 711.0 (88.8)	0.004
Female	32 105 (11.1)	28 526 (11.0)	30 384 (11.8)		28 709.9 (11.1)	3381.5 (11.2)	
Race and ethnicity^a^							
Black	47 856 (16.6)	42 852 (16.6)	5004 (16.5)	0.01	42 808.8 (16.6)	4890.8 (16.3)	0.02
White	194 213 (67.2)	173 703 (67.2)	20 510 (67.5)		173 809.2 (67.3)	20 205.3 (67.1)	
Other^b^	19 455 (6.7)	17 496 (6.8)	1959 (6.4)		17 409.2 (6.7)	2020.7 (6.7)	
Not known	27 334 (9.5)	24 423 (9.4)	2911 (9.6)		24 399.1 (9.4)	2975.7 (9.9)	
Aged ≥65 y	141 340 (48.9)	124 411 (48.1)	16 929 (55.7)	0.15	126 472.9 (48.9)	14 766.8 (49.1)	0.003
ASA Physical Status Classification >2	207 078 (71.7)	183 061 (70.8)	24 017 (79.0)	0.19	185 252.6 (71.7)	21 619.8 (71.8)	0.004
Surgical specialty							
ENT	11 180 (3.9)	10 804 (4.2)	376 (1.2)	0.46	10 008.0 (3.9)	1140.4 (3.8)	0.02
General	103 539 (35.8)	96 434 (37.3)	7105 (23.4)		92 638.5 (35.8)	10 763.8 (35.8)	
Neurosurgery	17 157 (5.9)	14 834 (5.7)	2323 (7.6)		15 341.8 (5.9)	1815.1 (6.0)	
OB/GYN	7593 (2.6)	6738 (2.6)	855 (2.8)		6794.4 (2.6)	804.2 (2.7)	
Orthopedics	95 367 (33.0)	80 447 (31.1)	14 920 (49.1)		85 314.2 (33.0)	10 001.0 (33.2)	
Peripheral vascular	32 653 (11.3)	29 602 (11.5)	3051 (10.0)		29 221.4 (11.3)	3403.4 (11.3)	
Plastic	5980 (2.1)	5504 (2.1)	476 (1.6)		5345.6 (2.1)	568.7 (1.9)	
Podiatry	7000 (2.4)	6635 (2.6)	365 (1.2)		6260.0 (2.4)	720.5 (2.4)	
Thoracic	7550 (2.6)	6850 (2.7)	700 (2.3)		6755.0 (2.6)	790.2 (2.6)	
Transplant	839 (0.3)	626 (0.2)	213 (0.7)		747.4 (0.3)	85.2 (0.3)	
Clean wound classification	213 591 (73.9)	189 736 (73.4)	23 855 (78.5)	0.12	191 116.4 (74.0)	22 367.8 (74.3)	0.009
Antibiotics use 30 d prior to surgery	25 736 (8.9)	21 259 (8.2)	4477 (14.7)	0.21	22 989.6 (8.9)	2659.6 (8.8)	0.002
Preoperative urinary retention	7984 (2.8)	6428 (2.5)	1556 (5.1)	0.14	7137.5 (2.8)	827.9 (2.8)	0.001
History of UTI	4969 (1.7)	3920 (1.5)	1049 (3.5)	0.13	4452.2 (1.7)	519.9 (1.7)	<0.001
Preoperative urinary catheterization	1131 (0.4)	871 (0.3)	260 (0.9)	0.07	1006.4 (0.4)	121.3 (0.4)	0.002
CCI	2.26 (2.48)	2.23 (2.47)	2.46 (2.53)	0.09	2.26 (2.48)	2.26 (2.47)	0.001

Abbreviations: ASA, American Society of Anesthesiologists; CCI, Charlson Comorbidity Index; ENT, ear, nose, throat; OB/GYN, obstetrics and gynecology; SMD, standardized mean difference; UTI, urinary tract infection.

^a^ Race and ethnicity data were obtained from the Clinical Data Warehouse, which reflects data entered into patient electronic medical records.

^b^ Other category included American Indian or Alaska Native, Asian or Pacific Islander, Hispanic Black, and Hispanic White.

Standardized mean differences (SMDs) were used to assess balance of covariates (both continuous and categorical) among groups. We decided a priori that covariates with an SMD higher than or equal to 0.20 after balancing would be included in the outcome regression for doubly robust estimation; however, doing so was not needed because all covariates were balanced.^19^ The same process was used for the implant-related analysis. Weighted logistic regressions were used to estimate the ORs for the respective outcomes based on urine culture. Analyses were performed between January 2023 and January 2024 using R, version 4.0.5 (R Core Team),^20^ including twang, version 2.5.^21^

## Results

The study population comprised 250 389 VA enrollees who underwent 288 858 surgical procedures, after excluding 301 patients for current UTI, 8425 patients for fever within 30 days of surgery, 263 for prior cystectomy, and 3 for missing data (Figure). Demographics of surgical patients were typical for VA population studies, with 88.9% (256 753) of surgical procedures received by males and 11.1% (32 105) by females; 48.9% (141 340) received by patients 65 years or older; and 16.6% (47 856) received by patients of Black, 67.2% (194 213) of White, and 6.7% (19 455) of other race and ethnicity (Table 1). Before IPTW, patients with a preoperative urine culture were older, more likely to have preoperative urinary retention and history of UTI, more likely to receive antibiotics within 30 days before surgery, and more likely to have a higher American Society of Anesthesiologists Physical Status Classification (Table 1). After IPTW, the patient population was well balanced (ie, all SMDs were <0.20) across all characteristics.

**Figure.**
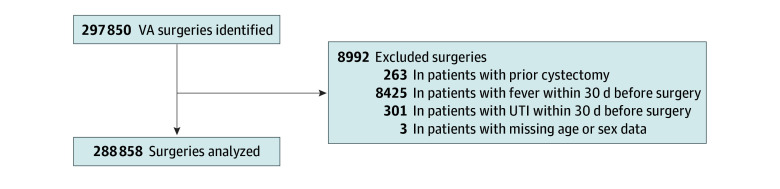
Flow Diagram of Study Population UTI indicates urinary tract infection; VA, US Department of Veterans Affairs.

Overall, preoperative urine culture was performed within 30 days before surgery for 10.5% of operations (30 384 of 288 858). The frequency of SSI was 1.3% (400 of 30 384) among surgical procedures for which a preoperative urine culture was performed and 1.5% (3837 of 258 474) among surgical procedures without such culture. In the analysis of all eligible surgical procedures, there were no significant differences in SSI when comparing those with vs without a preoperative urine culture (adjusted OR [AOR], 0.99; 95% CI, 0.90-1.10) (Table 2).

**Table 2.** Risk of Urinary Tract Infection (UTI) and Surgical Site Infection (SSI) Associated With Preoperative Urine Culture Performed in Surgical Patients

Balanced study population	Independent factor	Postoperative outcome	AOR (95% CI)
All eligible surgical procedures	30-d Preoperative urine culture performed vs not performed	SSI	0.99 (0.90-1.10)
		UTI	1.18 (0.98-1.40)
Orthopedic and neurosurgery	30-d Preoperative urine culture performed vs not performed	SSI	0.93 (0.76-1.12)
		UTI	1.27 (0.97-1.65)

Abbreviation: AOR, adjusted odds ratio.

Similarly, 0.6% of surgical procedures (173 of 30 384) for which a preoperative urine culture was performed were followed by a postoperative UTI compared with 0.4% of surgical procedures (1012 of 8474) without preoperative urine cultures. There were no significant differences in postoperative UTI in operations with a preoperative urine culture vs those without (AOR, 1.18; 95% CI, 0.98-1.40) (Table 2). Additionally, in the analysis that used the same methods but was limited to orthopedic surgery and neurosurgery, there was no significant difference in SSI (AOR, 0.93; 95% CI, 0.76-1.12) and postoperative UTI (AOR, 1.27; 95% CI, 0.97-1.65) between surgical procedures with and without preoperative urine cultures (Table 2).

## Discussion

In this analysis of nearly 290 000 surgical procedures in the national VA health care system, baseline characteristics were balanced to reduce bias associated with the routine selection of recipients of preoperative urine culture. With this robust analytical approach, we found that urine culture performance was not associated with decreased risk of UTI or SSI. The results also demonstrated no reduction in postoperative UTI or SSI risk by performing preoperative urine cultures in orthopedic and neurosurgical patients, a group vulnerable to the frequent use of implants and substantial morbidity in the event of infection. The lack of benefit is a critical message for surgeons trying to reduce SSI rates in this high-risk, high-consequence patient group. Focus should be turned to other measures that decrease SSI.^22^

We found that in the VA health care system, 10.5% of nongenitourinary surgical procedures were preceded by a preoperative urine culture. A recent study that evaluated the frequency and cost of low-value preoperative tests among patients within the VA system found that urinalysis, a common low-value test that precedes urine culture, was performed before 55 122 low-risk operations in 1 year, costing the VA over $200 000 annually.^23^ Similarly, urine culture is a low-value test and adds to unnecessary costs.

The untoward outcomes of unnecessary urine culture are well-described in the literature.^24^ When ASB is identified, almost 75% of patients get antibiotics.^25^ Small randomized clinical trials of antibiotic treatment vs no treatment of ASB primarily in nonsurgical populations have found more harm than benefit.^26,27^ A review of over 1600 patients found that antibiotic treatment of ASB was associated with resolution of the bacteriuria but not decreased symptomatic UTI, length of stay, or mortality.^26^ In addition, adverse events associated with the antibiotics, including antibiotic resistance, were substantially increased in treated patients. In an observational study of 729 patients with ASB conducted in the VA health care system, antibiotic treatment did not reduce readmission or all-cause mortality; *Clostridioides difficile* infection occurred in 0.4% without antibiotics vs 0.8% with antibiotics (not powered for significance).^25^ Additionally, previous work focusing on cardiac, vascular, and orthopedic surgical procedures reported that preoperative ASB was not associated with lower postoperative risk of infection, even when accounting for antibiotics.^6^ The current study moves the evidence base 1 step further by addressing the performance of the culture (as opposed to the result of the culture) in a broad range of presurgical patients.

To our knowledge, this study is the largest to date to evaluate the association between preoperative urine culture and outcomes. It included 10 different surgical specialties, including orthopedic surgery and neurosurgery. Even with the large sample size of surgical procedures from 112 hospitals over 3 years, we did not find any associations between performance of a preoperative urine culture and improved outcomes. Testing for ASB and unnecessary antibiotic use can be categorized as an assurance behavior; that is, performing additional service of marginal or no medical value in an attempt to avoid adverse outcomes and perform better on quality metrics. A large survey of surgeons found that 57% reported often ordering more tests than medically indicated and 36% reported prescribing more medications, such as antibiotics, than medically indicated.^28^ However, data of the current study showed that routine preoperative urine cultures among asymptomatic patients were not associated with reduction in adverse outcomes or improvement in quality metrics.

### Strengths and Limitations

The major strength of this study is the ability to balance the observed baseline characteristics between patients who did and did not receive urine culture. The main limitation of this study is its inability to control for unobserved and unobservable confounders. Despite controlling for a multitude of variables in the propensity score estimation, it is possible that residual confounding introduced bias into the regression estimates. There may be residual selection bias as we had information on only patients who underwent surgery, in contrast to a prospective trial that would still capture data from participants whose surgery was delayed or cancelled after urine culture was performed. Although of low frequency, procedures performed outside of the VA system and not captured in the electronic medical record were missing from the current study’s data. The absolute number of females evaluated in the study was small (32 105 [11.1%]) compared with males, making subgroup analysis by sex infeasible. In the neurosurgery and orthopedic surgery cohort, patient outcomes were not obtained beyond 30 days although the Centers for Disease Control and Prevention definitions allows 90 days of follow-up for infections after procedures with implants. Although the analysis approach reduced confounding, additional studies, including prospective clinical trials in different or more focused populations, are warranted.^29^

## Conclusions

In this cohort study of veterans undergoing noncardiac, nongenitourinary surgical procedures, we balanced factors, such as UTI history and patient comorbidities to address the concerns raised by clinicians that their patient is different or that studies have not accounted for the confounders in urine cultures and subsequent antibiotic treatment in this population. Despite data and guidelines, deimplementation of ingrained practices is challenging. The findings from this study can inform educational interventions and can be used for audit and feedback interventions to deimplement routine, unnecessary urine cultures and antibiotic treatment. Testing for ASB was not associated with reduced risk of SSI or UTI among the surgical specialties evaluated in this study. The results can change the potentially harmful practice of urine testing and antibiotic use in asymptomatic patients prior to surgery.

## Notes

Supplement.
Data Sharing Statement
