# Supplementary material for: Propensity Score-Weighted Analysis of Postoperative Infection in Patients With and Without Preoperative Urine Culture
Source: JAMA Netw Open. 2024 Mar 4;7(3):e240900. doi: 10.1001/jamanetworkopen.2024.0900 (PMC10912952; doi:10.1001/jamanetworkopen.2024.0900)
Supplement: Supplement. — Data Sharing Statement [file jamanetwopen-e240900-s001.pdf]

# Data Sharing Statement

O'Brien. Propensity Score-Weighted Analysis of Postoperative Infection in Patients With and Without Preoperative Urine Culture. *JAMA Netw Open*. Published March 04, 2024. doi:10.1001/jamanetworkopen.2024.0900

**Data**
**Data available:** No
